# Supplementary material for: Stigma, coping strategies, and their impact on treatment and health outcomes among young men living with HIV in Vietnam: A qualitative study
Source: PLOS Glob Public Health. 2022 Sep 20;2(9):e0000669. doi: 10.1371/journal.pgph.0000669 (PMC10021752; doi:10.1371/journal.pgph.0000669)
Supplement: Attachment — Submitted filename: Response to reviewers.docx [file pgph.0000669.s001.docx]

We appreciate the reviewers’ thoughtful evaluation of our manuscript *Stigma, coping strategies, and their impact on treatment and health outcomes among young men living with HIV in Vietnam: A qualitative study*. We have addressed each comment below, point-by-point, and have followed the specified journal style throughout the manuscript.

**Editor’s comments**:

1. Your co-author, Mary Bachman DeSilva - [mdesilva1@une.edu](mailto:mdesilva1@une.edu), has not confirmed authorship of the manuscript. We have resent them the authorship confirmation email; however please check that the above email address for them is correct and follow up personally to ensure they confirm.

*Response: Mary Bachman DeSilva confirmed her authorship of the manuscript on April 5, 2022.*

2. We do not publish any copyright or trademark symbols that usually accompany proprietary names, eg (R), (C), or TM  (e.g. next to drug or reagent names). Therefore please remove all instances of trademark/copyright symbols throughout the text, including TM on page 7.

*Response:* *We removed the trademark symbol “TM” .*

Additional Editor Comments (if provided):

3. Thank you for this submission. We see great promise in the work, but significant revisions are necessary to meet the criteria of the journal and reviewers. Most importantly, deeper engagement with the relevant literature on HIV, sexuality, and gender in contemporary Vietnamese society is essential. Further, clarity on qualitative methods, sample, and greater representation of the depth of qualitative data gathered.

*Response:* *Regarding the Methods section, our response follows Reviewer #2’s comments below. We appreciate the reviewer’s suggestions regarding the literature review. We have revised this section extensively, and have expanded our consideration of relevant research on HIV, sexuality and gender in global, low- and middle-income countries, and in Asia and Vietnam in response to your and Reviewer #1’s comments and on coping strategies for dealing with HIV and stigma in response to Reviewer #2’ comment.*

**Reviewer #1 comments:**

4. Reviewer #1: This article presents qualitative data on the experience of stigma among members of an understudied and undercounted, but nonetheless quite important population: young men living with HIV in Vietnam. Its findings offer a useful contribution to future public health interventions. For the most part, it is well prepared, logically laid out, and clearly written. Its methods are very clearly described. And despite the small N, which the authors acknowledge, the work presents important findings.

*Response: We thank you for your comment.*

Suggestions for revision:

5. Probably my most major suggestion: I would like to suggest that the authors consider revising this manuscript by developing a deeper engagement with the relevant literature on HIV, sexuality, and gender in contemporary Vietnamese society. There is very little by way of a literature review in this essay. At the same time, there is quite an extensive research base on these topics that has not been consulted fully; as a result, the study findings feel somewhat thinly contextualized. I would recommend that the authors read published works by Le Minh Giang, Alfred Montoya, Pauline Oosterhoff, and others, and actively include these in the literature review as points of reference.

*Response: Thank you for the suggestions and for the recommendations of specific authors. As noted in our response to the editor above, we have revised this section extensively, and have expanded our consideration of relevant research on HIV, sexuality and gender, and coping strategies. We reviewed the literature of Le Minh Giang, Alfred Montoya, Pauline Oosterhoff, and others, and have added references number 34, 35, 45 from Le Minh Giang, and references number 37, 38 from Alfred Montoya and other researchers as appropriate.*

6. Secondarily, I might also note that many of the quotes are very short and do not give a strong sense of the respondents' circumstances or worldview. Given that this is a qualitative study, it would be meaningful to have more expanded interview quotations. Perhaps it would be possible to expand some of these quotes.

*Response: We have expanded the quotations where it is possible, and the changes are all tracked in the revised Findings section.*

7. It would be useful to have a stronger sense re: whether these are entirely novel findings, or whether similar trends have been observed in other parts of the world. For example, on p. 19 the authors write that "social life with romantic partners is reduced significantly due to fear of transmission." Is this found in other global settings?

*Response:* *Thank you for the comment. In the revision of our Discussion, we have attempted to contextualize our findings more concretely. Regarding the finding "social life with romantic partners is reduced significantly due to fear of transmission.", in the Discussion section, we compared our observation with previous literature as follows:*

*“With romantic relationships and health care (apart from HIV care), avoidant coping strategies were more common. Similar dynamics have been observed in studies with adolescents and young adults living with HIV in the United States and in the United Kingdom (23,55). As with non-disclosure, avoidance of close relationships may reduce social support, and further impede mental wellbeing caused by social isolation.”*

*We also added comparisons with prior literature in one more place as follow:*

*“For MSM, HIV stigma does not exist in isolation, but coincides with the widespread stigma towards this population group in Vietnamese culture. The dual stigmatization of homosexuality and HIV and the common practice of non-disclosure have been observed in other studies with MSM living with HIV in Vietnam (31,46).”*

8. I might note that it would be useful to establish the ethnic identity and/or the socioeconomic status of the youth respondents, as this is not made clear and may supply useful context

*Response: We regret that we did not collect information about ethnicity. We added more information about participants’ socioeconomic status (i.e., employment status).*

9. It seems that there is a bit of a discrepancy between the statement that "the experience of internalized stigma appeared somewhat dependent on (...) personal preparedness" (9) and the interview quote that follows it on p. 10. That quote is interpreted as suggesting that it is not so much personal preparedness, but the youth's sense of not being responsible, that limited his experience of stigma. Perhaps this discrepancy could be resolved so the findings are better described.

*Response: We deleted this statement and moved the sentence about the youth who says that he contracted HIV by accident to the section on Internalized stigma as it reflects thoughts about the sense of self rather than the psychological impact when learning HIV status:*

10. I would suggest that the authors' finding regarding the possible need for better infection control protocols in health care --given the possibility that some HIV+ individuals, like the one quoted interview respondent, may not disclose HIV status to care providers -- is a bit of a tangential finding. While of potential importance, it should be subordinated to the findings that *directly* address the topic of stigma. Listing it as finding #2 of 3 findings somewhat distracts from the focus of the research.

*Response: We agree with this observation, and have omitted this point from both the Abstract and the Discussion.*

11. Finally, there are some line-level instances of unclear language or typographical errors, etc.:

*Response: We have corrected all typographical errors in the manuscript, and have revised specific instances of unclear language as below:*

a. "their highly perceived stigma resulted in not fully ..." (11) - rephrase for clarity

*Response: We revised as follows:*
 *“All participants revealed to an extent that they did not experience much if any interpersonal stigma, because their high degree of perceived stigma resulted in limited disclosure—to family, friends, peers, and/or within the workplace or healthcare setting.”*

b. "PHUC-blessed" (12) - Vietnamese language does not need to be in all caps (and usually is set in italics and [square brackets] "good luck or 'PHUC'" (12) - same as above

*Response: We revised as suggested.*

c. "oreover" (12) - Should be "Moreover"

*Response: We revised.*

d. "a social evil" (12) - This should be explained and further discussed in a footnote or a parenthetical comment, as many readers will not be familiar with the concept of "te nan xa hoi" in Vietnam

*Response: We think that it is important to provide this information as a context of HIV, gender, sexuality situation in Vietnam, so we provide this information in Introduction as below:*

*“The experience of men living with HIV in Vietnam is affected by both societal perceptions of HIV and attitudes toward MSM (42,43). In the 2000’s, homosexuality was considered to be a “social evil” (tệ nạn xã hội) by the government (44)*. *Social evils “run counter to the morality and fine customs and habits of the nation, adversely affect the health of the race, and the material and cultural life of The People” (45).” However, due to rapid socioeconomic transformation, knowledge of and attitude towards homosexuality have improved, and expressions of sexual identity are more liberated. Nonetheless, stigma towards homosexuality remains common. Drug use, prostitution, and gambling are also considered social evils in Vietnamese culture. PLHIV are often assumed to be drug users or involved with sex work and/or homosexuality (34).*”

e. "MSM who may want multiple sexual partners" (13) - maybe this could be better described (it's unclear whether the MSM in question may want multiple sexual partners simultaneously or sequentially)

*Response: Thank you for your feedback.* *We have revised the quotation for clarity as below:*

*“Some patients shared with us that they had so many partners that they couldn’t remember them all. Regarding the characteristics of MSMs, unlike women who usually talk with partners to get to know them better before going out, MSM often go to various websites to search for sexual partners. If the sex is good enough, they will make another date. If not, after that sexual encounter, they will not contact the person again. They also usually contact each other by their fake name or nickname only…If they say they have HIV, they are afraid that their partners will not love them anymore, which means no more sexual partners. (Doctor)”*

f. On p. 17 there is a paragraph discussing "less than half (4/9) participants revealed involvement with a romantic partner" and "of the seven single participants, most (4/7) indicated an active avoidance of personal relationships" -- the numbers are not adding up fully clearly here, and too, "personal relationships" is not clear. Does this mean "sexual or romantic relationships"?

*Response: We revised as below:*

*“Except for one participant who characterized himself as asexual, less than half (4/9) participants revealed involvement with a romantic partner*. *Just over half (5/9) indicated an active avoidance of romantic relationships.*”

g. On p. 20, the respondent is quoted saying "I revealed my gender," but I believe this should be translated as "I revealed my sexual orientation."

*Response: Thank you, we revised it.*

h. On p. 22: "interations" instead of interactions

*Response: We revised it.*

i. On p. 23: "motorcyle" instead of motorcycle

*Response: We revised it.*

j. On p. 26: "The fear of rejection and discrimination ... was severe" - How is "severe" being defined?

*Response: As this is qualitative study, we do not have a definition for “severe” in this context, so we revised from “severe” to “significant”.*

k. On p. 26: "descendents" instead of descendants

*Response: we revised it.*

l. On p. 26: "in previous studies" - they should be cited here

*Response: We added references as below:*

*“Quite different from the experiences found in previous studies (25,26)”*

m. On p. 26: "in light of their gender" instead of "in light of their sexual orientation"

*Response: We revised it.*

n. On p. 26: "maintain face for the family and have descendents" - the second quotation mark (") is missing

*Response: We revised it.*

o. On p. 27: "adversedly" instead of adversely

*Response: We revised it.*

p. On p. 27: Reference #14 does not appear appropriately scholarly and should be replaced.

*Response: We believe this source Personality Psychology: Domains of Knowledge About Human Nature* *is appropriate. The textbook contains a separate section about disclosure/ non-disclosure as coping strategies in human behavior. We added more information about this section in the list of references and have also added a reference for the effect of stigma on mental health as below:*

*“These findings are consistent with research which has found that non-disclosure is stressful and can worsen mental health (25) which is already negatively affected by internalized and perceived stigma (3).”*

*After adding more literature review, this reference number now is 25:*

*Larsen R, Buss D. “Disclosure”. Chapter 18. Stress, Coping, Adjustment and Health. Personality Psychology: Domains of Knowledge About Human Nature. 6th ed. McGraw-Hill Education; 2020.*

q. On p. 27: "advertenly" - should be intentionally

*Response: We revised it.*

r. On p. 27: "The YLHIV who supported his mother to cope with his HIV status did showed the same approach" - should be used the same approach

*Response: we revised it as below:*

*“The YLHIV who supported his mother as she learned to cope with his HIV status demonstrated exactly the same approach.”*

s. On p. 27: "todisclose" - should be to disclose

*Response: We revised it.*

t. On p. 28: "the published evidence base" - which evidence specifically is being cited?

*Response: We revised as below:*

*“As a framework for developing further research in this area, we developed a model based on our findings and the published evidence base (19–24,26–28).”*

u. On p. 28: "a descendent" - should be a descendant

*Response: We revised it.*

v. On p. 29: Limitations should include a comment about the study being cross-sectional, only drawing data from a single point in time

*Response: We revised as below:*
*“First, this is a cross-sectional study so the findings only reflect the experiences of the participants as reported at a single point in time.”*

w. On p. 29: "the results likely reflect the situation of young MSM more than other groups" - it would make sense to rephrase this, because the results *definitely* reflect the situation of young MSM and not that of other groups

*Response: We revised as below:*

*“Second, since nearly all of the participants were MSM, the results mainly reflect the situation of young MSM.”*

x. References - As stated, I am not sure that reference #14 appears adequately scholarly. Again, the references appear to be on the limited side and to feature very little from the extensive public health and social science scholarship on Vietnam. These works and their findings should be incorporated and should inform the arguments presented in this essay.

*Response*: *We explained about reference #14 in the comment # 11p. Regarding the point about literature review, we addressed this in our response to the Editor’s comment number #1.*

**Reviewer #2: Comments to the Authors**

This manuscript explores Stigma, coping strategies, and their impact on treatment and health outcomes among young men living with HIV in Vietnam. These data are important. Below are some comments which will hopefully help the authors in strengthening their manuscript.

Introduction

12. Overall:

There is a lack synthesis of literature globally, in context of Asia and Vietnam about HIV-stigma towards YLHIV. Similarly, there is a lack synthesis of the existing literature on HIV-related coping strategies that have been used by YLHIV. Authors need to provide these in order to support their claim about the gap in knowledge they are trying to bridge with this study.
*Response: We added a synthesis of the literature about HIV-stigma globally and in context of Asia and Vietnam as described in our response to the editor’s comment #1. We also added a review of literature on HIV-related coping strategies.*

13. Authors really need to provide clear definition of these terms: enacted stigma (it is about both stigmatising and discriminatory attitudes and behaviours/acts); perceived stigma; and internalised/self-stigma.

*Response:* *We have provided the definition of 3 types of stigma in the first paragraph of the introduction.*

14. Introduction. I suggest these current publications to be looked at:

1. HIV Stigma and Moral Judgement: Qualitative Exploration of the Experiences of HIV Stigma and Discrimination among Married Men Living with HIV in Yogyakarta
2. Stigma and Discrimination towards People Living with HIV in the Context of Families, Communities, and Healthcare Settings: A Qualitative Study in Indonesia

*Response:* *We have reviewed the above publications and included them in our literature review as references number 6 and number 7 as below:*

*“Studies have documented that PLHIV in resource-limited settings like Vietnam, Indonesia, and Thailand experience stigma and discrimination from families, communities and health care settings (5–9).”*

15. Also, be consistent in using these terms throughout the manuscript.

*Response:* *We revised to be consistent in using “enacted stigma”, “perceived stigma” and “internalized stigma”*

16. “The prevalence of adverse mental health outcomes among YLHIV is higher than their counterparts not living with HIV”
    • What is the percentage or number? Provide it.

*Response:* *As there is no meta-analysis available yet, we provide the percentage in 2 studies as below:*

*“Adverse mental health outcomes are prevalent among YLHIV (10–13), and have generally been found to be substantially higher than among counterparts not living with HIV across contexts in the few studies where comparison groups are included (10). For example, a study in the United States found that 21% of YLHIV reported mood disorders, significantly higher than the 5% of youth not living with HIV (14). In Zambia, adolescents living with HIV had a significantly higher prevalence of mental health problems (29.1% compared to 16.5% in a community comparison group), particularly emotional symptoms (31.5% vs. 11.2%) and peer problems (41.8% vs. 9.2%) (15).”*

17. “The fact that new HIV infections are rising among men in young age groups is
    troubling, and may foreshadow further problems related to stigma”

• What is the age range?
• What is the percentage/ number new HIV infection that is rising?

*Response:* *We deleted the sentence. However, we still provided the information about the age range and the percentage where appropriate as below:
“40.1% of new HIV infections in Vietnam occur among individuals 16-29 years old (28), and MSM represent a large percentage of new infections.* *In 2015, HIV sentinel sero-surveillance estimated the HIV prevalence among MSM in Vietnam to be 5.2%; by 2017, prevalence had doubled (12.2%) (30). HIV prevalence among MSM in Hanoi specifically increased from 9.4% in 2006 to 20% in 2010 (31).”*

18. The extent and experience of stigma among younger men living with HIV is not well
    documented, however, especially in limited resource settings and in Asian cultures, where HIV-related stigma is particularly pervasive in part due to societal emphasis on the collective and cultural values (9).

• Authors need to provide more references about stigma in other Asian countries to support their claim that stigma is pervasive within communities in general in Asia
• You can use two sources above, and in addition, this one: “HIV stigma and discrimination: perspectives and personal experiences of healthcare providers in Yogyakarta and Belu, Indonesia”
 *Response:* *As described above, we have substantially revised the introduction and literature review, including expanding the evidence cited for stigma research in other Asian countries included the suggested references as below:*

*“Studies have documented that PLHIV in resource-limited settings like Vietnam, Indonesia, and Thailand experience stigma and discrimination from families, communities and health care settings (5–9).”*

19. Data Collection

Who were the interviewers? Were they some of the authors? Authors need to be transparent about this. I suggest the authors to use COREQ checklist to guide the report of the methods section.

*Response: We appreciate these suggestions, and have incorporated them into our elaboration of the methods section:*

*“Two female interviewers with background in social science research methods and experience working with PLHIV conducted IDIs in Vietnamese in private rooms at the clinic. The first author was one of these interviewers.*

20. What was the range duration of the interviews?

*Response: We provide the information as below:*

*“Interviews lasted from 40 to 60 minutes and were audio-recorded and supplemented with written notes.”*

21. How did you decide the number of participants in your study? Whether data saturation was reached? How did you determine data saturation?

*Response:* *We revised to provide more information on the sampling as below:*

*“The qualitative study was conducted as part of the development process for a stigma reduction intervention, and the number of interviews was determined in large part by time and grant resources available.  Given that common themes recurred frequently throughout interviews, we are confident that we came reasonably close to data saturation, whereby no further major themes were likely to be discovered (48).”*

22. Analytic Method

Who did the transcription, translation and coding or data analysis? Were they the interviewers?
Authors need to provide information about the role of the authors in this research.

It is indicated that interviews were conducted by multiple interviewers. How do you solve any discrepancies, if any, in your interpretation about the data during data analysis?

What qualitative data analysis framework the authors used to guide a step-by-step of data analysis which led to the final results (themes or categories) presented in the finding section?

Was the analysis deductive or inductive or both?

*Response: We have added additional detail regarding our approach to data analysis, as well as roles of members of the research team as below:*

*“Audio-recordings were transcribed in Vietnamese by two researchers, one of whom was an interviewer.The transcripts and data interpretation were reviewed by both interviewers.  When discrepancies were identified, the researchers consulted the Vietnamese transcripts together until consensus was reached regarding interpretation. The transcripts were then translated into English by a bilingual translator.* *English transcripts were reviewed by a second investigator for accuracy, then coded by one of the authors (JS) and analyzed using NVivo software and an iterative codebook [(50)]. We used a thematic content analysis approach to identify important themes present in the transcripts. We built an interative codebook starting with the broad domains of inquiry as outlined in the interview guide, then as transcripts were coded in NVivo, continued to identify and create additional codes as they emerged in individual transcripts.  After initial coding of all transcripts, the coder re-visited each transcript and applied all relevant codes from the comprehensive codebook that was developed in the process. Preliminary analysis revealed major themes, with further generation of sub-themes. We categorized responses by their frequency and also considered contrasting observations. The approach was primarily inductive and arose from the data, although some deductive elements were present, including initial broad domains as queried in the interview guide.“*

23. Findings

Overview
What is the rationale behind choice of only male youth in this study?

*Response:* *The rationale is based on the fact that young men represent the largest proportion of new HIV infections, and are considered a priority group for the national HIV prevention and treatment program in Vietnam. We have added the rationale in the Introduction as below:*

*“In Vietnam, approximately 215,000 people were living with HIV in 2019 (29), and although new HIV infections and AIDS-related deaths have plateaued in recent years in the country, the number of PLHIV is estimated to be 44 times greater than reported (30). 40.1% of new HIV infections in Vietnam occur among individuals 16-29 years old (29), and MSM represent a large percentage of new infections.*

24. General Experience of Stigma
    Internalised Stigma
    The first two paragraphs under "Internalised Stigma" section are not about internalised stigma but psychological/mental health impacts of HIV. These need to be revised.

    Internalised/self-stigma refers to the degree to which PLHIV endorse negative perceptions, beliefs, feelings associated with HIV and apply these to themselves.

    I suggest the authors to provide clear definitions about internalised/self-stigma, perceived stigma and enacted stigma in the introduction and use them to guide the presentation of their findings. Your narratives/synthesis and the quotes that support your synthesis should reflect these concepts.

*Response:* *Thank you for your comments. For clarity, we have divided the section into 2 parts: 1) psychological impacts when learning HIV status; and 2) internalized (self) stigma*.

25. Enacted stigma

No need to report as it was not experienced by your participants.

*Response*: *We believe that it is notable that participants did not experience enacted stigma, and thus have chosen to report this.*

26. Dynamics underlying internalised stigma and perceived stigma

Again, this section is mainly about HIV-related psychological challenges/impacts participants experienced.

Authors need to carefully look at this and revise, probably combine it with the first two paragraphs in the internalised stigma section under a new section about psychological impacts of HIV.
*Response:* *The definition of internalized stigma which we used in this study is “the incorporation of the devalued attribution of society into one’s sense of self.” And the definition of perceived stigma is individual’s subjective perception of their stigmatized status.*

*Regarding quantitative measurement, internalized stigma is measured by questions about self-blaming, guilty, low self-esteem, suicidal thoughts… related to HIV status. Therefore, internalized stigma is overlap with the psychological impacts of HIV.*

*We choose the title of this section is “Dynamics underlying internalized stigma and perceived stigma” because it covers individual/ family/social factors contributing to their negative attribution of HIV status and the perception of YLHIV about society attitude towards their HIV status. We hope that our explanation clarifies your concern.*

27. Discussion

Authors discussed the findings very well

*Response: We thank you for your comment.*

28. Limitations

Generalisability is not an aspect to be achieved in qualitative research (it is typically for quantitative research), so it is not a limitation of qualitative studies.

*Response:* *We deleted the limitation about generalizability.*
