# Supplementary material for: Stigma, coping strategies, and their impact on treatment and health outcomes among young men living with HIV in Vietnam: A qualitative study
Source: PLOS Glob Public Health. 2022 Sep 20;2(9):e0000669. doi: 10.1371/journal.pgph.0000669 (PMC10021752; doi:10.1371/journal.pgph.0000669)
Supplement: Attachment — Submitted filename: Response to reviewers.docx [file pgph.0000669.s002.docx]

**Journal Requirements:**

1. Please review your reference list to ensure that it is complete and correct. If you have cited papers that have been retracted, please include the rationale for doing so in the manuscript text, or remove these references and replace them with relevant current references. Any changes to the reference list should be mentioned in the rebuttal letter that accompanies your revised manuscript. If you need to cite a retracted article, indicate the article’s retracted status in the References list and also include a citation and full reference for the retraction notice.

*Response: We have reviewed the reference list to make sure it is complete and correct.*

**Reviewer #2 comments:**

Overall, I am happy with the revision provided by the authors. Just a few small suggestions to add and can be published.

*Response: We thank you for your comment.*

Analytic methods
“We used a thematic ...... in the interview guide.”

Comment:
• I agree, but as you haven’t provided any reference, please use this current literature as a reference to support this, look at the data analysis section that applied same kind of analysis:

Cultural and religious determinants of HIV transmission: A qualitative study with people living with HIV in Belu and Yogyakarta, Indonesia. PLoS ONE 16(11): e0257906. <https://doi.org/10.1371/journal.pone.0257906>

*Response: Thank you. We regret the oversight and have now included a standard reference for thematic analysis from SAGE Publications as below:*

*Braun V, Clarke V. Thematic analysis: a practical guide to understanding and doing. 1st ed. Thousand Oaks: SAGE Publications; 2021*

Enacted stigma
All participants revealed ...healthcare setting.

Comments:
• I don’t agree with the inclusion of this ‘enacted stigma’ in your results section, but if you do want to present it in the results section, then it is not proper to jus write the above sentence. You need to walk the reader through the story why it was not the case in your current participants and support your narratives with quotes from your participants.

I suggest, you take it our from results section and address it in the discussion section to inform the readers why it was not the case in your participants

*Response: Thank you. We have removed ‘enacted stigma’ from the results section and have included it in the discussion section as below:*

*“Enacted stigma was less prevalent, as the high degree of perceived stigma resulted in non-disclosure or very limited disclosure to trusted persons, effectively minimizing opportunity for enacted stigma to occur.”*

Discussion
Quite different from the experiences found in previous studies (26,27),... sexual orientation as well as their serostatus.

Comment:
• I suggest the authors use this current literature review about stigma in Asia to support this:

Psychological and Social Impact of HIV on Women Living with HIV and Their Families in Low- and Middle-Income Asian Countries: A Systematic Search and Critical Review. Int. J. Environ. Res. Public Health 2022, 19, 6668. <https://doi.org/10.3390/ijerph19116668>

*Response: Thank you for the suggestion. We reviewed the recommended literature review and have added it as a reference number 51 as below:.*

*“Quite different from the experiences found in previous studies (26,27,51),Vietnamese YLHIV face other cultural pressures…”*

Limitations
“….this is a cross-sectional study…..”

Comments:
• Please be careful with the terms, “cross-sectional” is typical quantitative study design (not qualitative design). Please change it and just simply use “qualitative study”.

*Response:* *Thank you for your comment. We changed from “cross-sectional study” to “qualitative study”:*
